# The benefits of youth are lost on the young cardiac arrest patient

**DOI:** 10.12688/f1000research.9316.1

**Published:** 2017-01-25

**Authors:** Brian Griffith, Patrick Kochanek, Cameron Dezfulian

**Affiliations:** 1 Safar Center for Resuscitation Research, University of Pittsburgh School of Medicine, Pittsburgh, Pennsylvania, USA; 2 Department of Critical Care Medicine, University of Pittsburgh School of Medicine, Pittsburgh, Pennsylvania, USA; 3 Pediatrics, University of Pittsburgh School of Medicine, Pittsburgh, Pennsylvania, USA; 4 Clinical and Translational Sciences, University of Pittsburgh School of Medicine, Pittsburgh, Pennsylvania, USA; 5 Vascular Medicine Institute, University of Pittsburgh School of Medicine, Pittsburgh, Pennsylvania, USA

**Keywords:** Cardiac arrest, Paediatric, Resuscitation

## Abstract

Children and young adults tend to have reduced mortality and disability after acquired brain injuries such as trauma or stroke and across other disease processes seen in critical care medicine. However, after out-of-hospital cardiac arrest (OHCA), outcomes are remarkably similar across age groups. The consistent lack of witnessed arrests and a high incidence of asphyxial or respiratory etiology arrests among pediatric and young adult patients with OHCA account for a substantial portion of the difference in outcomes. Additionally, in younger children, differences in pre-hospital response and the activation of developmental apoptosis may explain more severe outcomes after OHCA. These require us to consider whether present practices are in line with the science. The present recommendations for compression-only cardiopulmonary resuscitation in young adults, normothermia as opposed to hypothermia (33°C) after asphyxial arrests, and paramedic training are considered within this review in light of existing evidence. Modifications in present standards of care may help restore the benefits of youth after brain injury to the young survivor of OHCA.

## Introduction

Outcomes by age in acute neurologic injuries tend to favor youth. When stratified by age, younger age groups tend to have improved rates of mortality and favorable neurologic recovery in traumatic brain injury (TBI) ^1– 3^ and stroke ^4– 6^, including both intracerebral ^7, 8^ and subarachnoid ^9^ hemorrhages. Children and young adults have fewer comorbidities and more reserve by virtue of their youth. It is well known that vascular disease increases with each decade of life ^10^ such that one would expect far better cerebral and coronary blood flow assuming that the same quality of cardiopulmonary resuscitation (CPR) was administered. Age-predicted functional and electroencephalogram recovery from intracarotid amobarbital ^11^ is consistent with the fact that younger brains are either more resistant to the insult or faster to recover. After TBI, younger patients compared with older ones with a similar disability score at admission to rehabilitation exhibited increased recovery after 1 and 5 years ^1^.

Indeed, this pattern of less mortality and disability with younger age is observed throughout critical care and medicine. Yet after out-of-hospital cardiac arrest (OHCA), outcomes are remarkably similar in both the short and long term when comparing pediatric and adult patients ( Table 1). This remains the case in nations where pre-hospital termination of resuscitation efforts is not permitted ( Table 2). Survival to hospital discharge and good neurologic function at discharge and 1 month are all below 10% in most studies. It is only when one considers long-term outcomes that children appear to have a slight advantage in outcome.

**Table 1.** Survival and favorable neurologic outcome rates following out-of-hospital cardiac arrest by age group.

Age group	Survival to hospital discharge, percentage (age, number)	Favorable neurologic outcome at discharge, percentage (age, number)	Survival to 1 month, percentage (age, number)	Favorable neurologic outcome at 1 month, percentage (age, number)	Long-term survival, percentage (age, time of follow-up, number)	Long-term favorable neurologic outcome, percentage (age, time of follow-up, number)
<1 year old Infants	9.5 (<1, 359) ^12^ 3.9 (<1, 232) ^13^ 8.9 (<1, 45) ^14^ 2.8 (<1 year old, 283) ^62^ Mean = 6.0 (919)	6.7 (<1, 45) ^14^	5.1 (<1, 363) ^15^ 5.2 (<1, 343) ^19^ Mean = 5.2 (706)	1.2 (<1, 343) ^19^	12.0 (<1, 1 year, 25) ^63^	
1 to 5 years old Preschool children	13.3 (1–4, 180) ^12^ 10.4 (1–11, 135) ^13^ 3.0 (1–11, 66) ^14^ 4.3 (1–11, 276) ^62^ Mean = 7.9 (657)	3.0 (1–11, 66) ^14^ 1.6 (1–8, 129) ^64^ Mean = 2.1 (195)	11.0 (1–4, 190) ^15^ 8.7 (1–4, 172) ^19^ Mean = 9.9 (362)	1.7 (1–4, 172) ^19^	34.8 (1–8, 1 year, 46) ^63^ 12.9 (0–18, 1 year, 101) ^65^ Mean = 19.7 (147)	16.2 (0–17, 1 year old, 260) ^30^ 5.9 (0–18, 1 year old, 101) ^65^ Mean = 13.3 (361)
6 to 12 years old School-aged	10.4 (1–11, 135) ^13^ 4.3 (5–12, 92) ^12^ 3.0 (1–11, 66) ^14^ 4.3 (1–11, 276) ^62^ Mean = 5.6 (569)		7.5 (5–12, 178) ^15^ 6.8 (5–12, 117) ^19^ Mean = 7.1 (295)	1.7 (5–12, 117) ^19^	27.4 (9–16, 1 year, 95) ^63^ 12.9 (0–18, 1 year, 101) ^65^ Mean = 19.9 (196)	16.2 (0–17, 1 year, 260) ^30^ 5.9 (0–18, 1 year old, 101) ^65^ Mean = 13.3 (361)
13 to 18 years old Adolescence	12.5 (12–19, 136) ^13^ 4.9 (12–20, 122) ^14^ 6.5 (12–19, 340) ^62^ Mean = 7.5 (598)	4.1 (12–20, 122) ^14^ 10.2 (9–17, 88) ^64^ Mean = 6.7 (210)	12.6 (13–21, 590) ^15^ 13.9 (13–17, 108) ^19^ Mean = 12.8 (698)	11.1 (13–17, 108) ^19^	27.4 (9–16, 1 year old, 95) ^63^ 12.9 (0–18, 1 year, 101) ^65^ Mean = 19.9 (196)	16.2 (0–17, 1 year old, 260) ^30^ 5.9 (0–18, 1 year old, 101) ^65^ Mean = 13.3 (361)
18 to 39 years old Young adult	9.0 (20–39, 665) ^66^ 8.8 (16–39, 1,406) ^17^ 10.5 (18–34, 1,164) ^67^ 7.8 (18–35, 197) ^69^ 11.4 (18–39, 928) ^54^ Mean = 9.8 (4,360)	~4.4 (18–64, 50,830) ^69^ 7.4 (18–44, 2,212) ^70^ ~20 (18–39, 76) ^71^ Mean ≈ 4.5 (53,118)	8.8 (22–35, 1,543) ^15^ 6.8 (18–35, 1,235) ^72^ 13.8 (18–64, ≈3,250) ^73^ 13.2 (18–65, 7,227) ^74^ 7.1 (19–29, NR) ^75*^ 3.9 (30–39, NR) ^75*^ Mean = 12.2 (13,255)		12.5 (18–64, 6 months, ≈3,250) ^73^	84 (18–39, 5 years old, 920) ^54^
40 to 64 years old Adult	10.4 (40–59, 2,858) ^66^ 12.2 (35–49, 4,154) ^67^ 12.5 (50–64, 9,547) ^67^ Mean = 12.1 (16,559)	2.4 (>55, 35,099) ^70^ ~24 (40–59, 192) ^71^ Mean ≈2.5 (35,291)	4.5 (40–49, NR) ^75*^ 4.3 (50–59, NR) ^75*^ 3.8 (60–69, NR) ^75*^		17.7 (51–70, 6 months, 198) ^76^	
65 to 80 years old Older adult	9.3 (60–74, 3304) ^66^ 8.5 (65–79, 4593) ^77^ 9.3 (65–79, 9,092) ^67^ 15.4 (70–79, 699) ^78^ Mean = 9.3 (17,688)	13.8 (70–79, 699) ^78^ ~1.8 (65–84, 107,945) ^69^ ~15 (60–79, 204) ^71^ Mean ≈1.9 (108,848)	3.2 (65–75, 7148) ^75^ 11.9 (65–74, ≈1,600) ^73^ 5.6 (66–80, 8,919) ^74^ Mean ≈5.2 (≈17,667)		11.0 (65–74, 180 days, ≈1,600) ^73^ 7.0 (71–90, 6 months, 199) ^76^ 13.7 (70–79, 699) ^78^ Mean ≈11.4 (≈2,498)	
>80 years old Elderly	4.5 (≥75, 3854) ^66^ 4.0 (≥80, 3,032) ^77^ 4.6 (≥80, 7,058) ^67^ 7.6 (≥80, 633) ^78^ Mean = 4.6 (14,577)	6.6 (≥80, 633) ^78^ ~0.6 (≥85, 60,717) ^69^ ~5 (≥80, 116) ^71^ Mean ≈0.8 (61,466)	11.9 (75–84, ≈2,400) ^73^ 10.9 (≥85, ≈1,550) ^73^ 2.5 (>75, 8,269) ^75^ 2.0 (>80, 5,334) ^74^ Mean ≈ 4.4 (≈17,553)		10.7 (75–85, 6 months, ≈2,400) ^73^ 10.1 (≥85, 180 days, ≈1,550) ^73^ 6.5 (≥80, 633) ^78^ Mean ≈ 9.9 (≈4,583)	

Data were extracted from multiple epidemiologic reports as cited. The denominator was emergency medical services-treated out-of-hospital cardiac arrest. In many cases, our age bands do not strictly conform to other studies; therefore, data may be repeated and actual age ranges are provided for each data element. The dotted line represents the separation of children and young adults (0–39 years old) from older adults (>40 years old). One study reported data in graphic form only; therefore, sample sizes are estimates (denoted by ≈). For each category, we calculated a mean across studies by pooling outcomes and denominators but excluding those that did not report samples size (denoted by *). NR, not reported.

**Table 2.** Survival and neurologic outcome rates following out-of-hospital cardiac arrest by age group in countries that do not follow pre-hospital termination of resuscitation protocols.

Age group	Survival to hospital discharge, percentage (age, number)	Favorable neurologic outcome at discharge, percentage (age, number)	Survival to 1 month, percentage (age, number)	Favorable neurologic outcome at 1 month, percentage (age, number)	Long-term survival, percentage (age, time of follow-up, number)	Long-term favorable neurologic outcome, percentage (age, time of follow-up, number)
<1 year old Infants	2.8 (<1 year old, 283) ^62^		5.2 (<1, 343) ^19^ = 19	1.2 (<1, 343) ^19^		
1 to 5 years old Preschool children	4.3 (1–11, 276) ^62^		8.7 (1–4, 172) ^19^	1.7 (1–4, 172) ^19^		
6 to 12 years old School-aged	4.3 (1–11, 276) ^62^		6.8 (5–12, 117) ^19^	1.7 (5–12, 117) ^19^		
13 to 18 years old Adolescence	6.5 (12–19, 340) ^62^		13.9 (13–17, 108) ^19^	11.1 (13–17, 108) ^19^		
18 to 39 years old Young adult		~4.4 (18–64, 50,830) ^69^ 7.4 (18–44, 2,212) ^70^	13.8 (18–64, ≈3,250) ^73^		12.5 (18–64, 6 months, ≈3,250) ^73^	
40 to 64 years old Adult		2.4 (>55, 35,099) ^70^				
65 to 80 years old Older adult		~1.8 (65–84, 107,945) ^69^	11.9 (65–74, ≈1,600) ^73^		11.0 (65–74, 180 days, ≈1,600) ^73^	
>80 years old Elderly		~0.6 (≥85, 60,717) ^69^	11.9 (75–84, ≈2,400) ^73^ 10.9 (≥85, ≈1,550) ^73^		10.7 (75–85, 6 months, ≈2,400) ^73^ 10.1 (≥85, 180 days, ≈1,550) ^73^	

Data were extracted from a subset of the epidemiologic reports in Table 1 (as cited). The denominator was emergency medical services-treated out-of-hospital cardiac arrest. In many cases, our age bands do not strictly conform to other studies; therefore, data may be repeated and actual age ranges are provided for each data element. The dotted line represents the separation of children and young adults (0–39 years old) from older adults (>40 years old). One study reported data in graphic form only; therefore, sample sizes are estimates (denoted by ≈). NR, not reported.

## Explanations for the lack of neurologic protection in younger out-of-hospital cardiac arrest

The consistent lack of witnessed arrests among pediatric OHCA, particularly in infants and preschool children, accounts for a substantial portion of the mortality ( Table 3). In the case of infants, most deaths occur during sleep and are attributed to sudden infant death syndrome (most of the “Other etiology” in infants on Table 3) or suffocation ^12– 15^. In preschool- and early school-aged children, asphyxia from drowning, choking, or suffocation ^15^ again occurs often out of immediate sight of an adult. Indeed, having OHCA at home is an independent predictor of worse outcome in some OHCA studies ^15^. In older children (for example, adolescents) and young adults, OHCA is often unwitnessed as it is the result of suicide or substance abuse with overdose ^12, 13, 15– 17^. A recent report from the Resuscitation Outcomes Consortium (ROC) documents the fact that increasing overdose deaths tend to occur in young adults and tend to be unwitnessed ^16^.

**Table 3.** Etiology and witnessed status of out-of-hospital cardiac arrest by age group.

Approximate age division	Cardiac etiology, percentage (age, number)	Respiratory etiology, percentage (age, number)	Other etiology, percentage (age, number)	Witnessed arrest, percentage (age, number)
<1 year old Newborn	12 (<1, 363) ^15^ 11 (<1, 276) ^72^ 39 (<1, 29) ^14^ 34 (<1, 347) ^19^ Mean = 20 (1,015)	6 (<1, 363) ^15^ 7 (<1, 276) ^72^ 37 (<1, 347) ^19^ Mean = 17 (986)	82 (<1, 363) ^5^ 82 (<1, 276) ^72^ 29 (<1, 347) = 19 Mean = 63 (986)	28 (<1, 363) ^5^ 26 (<1, 276) ^2^ 17 (<1, 277) ^3^ 10 (<1, 343) ^19^ Mean = 20 (1,259)
1 to 5 years old Preschool children	11 (1–4, 190) ^15^ 13 (1–4, 136) ^72^ 28 (1–11, 18) ^14^ 31 (1–4, 203) ^19^ Mean = 20 (547)	39 (1–4, 190) ^15^ 38 (1–4, 136) ^72^ 38 (1–4, 203) ^19^ Mean = 38 (529)	50 (1–4, 190) ^15^ 45 (1–4, 136) ^72^ 31 (1–4, 203) ^19^ Mean = 41 (529)	34 (1–4, 190) ^15^ 34 (1–4, 136) ^72^ 32 (1–11, 154) ^13^ 39 (1–8, 129) ^64^ 31 (1–4, 172) ^19^ Mean = 34 (781)
6 to 12 years old School-aged	13 (5–12, 178) ^15^ 14 (5–12, 142) ^72^ 30 (5–12, 135) ^19^ Mean = 18 (455)	30 (5–12, 178) ^15^ 30 (5–12, 142) ^72^ 37 (5–12, 135) ^19^ Mean = 32 (455)	57 (5–12, 178) ^15^ 56 (5–12, 142) ^72^ 33 (5–12, 135) ^19^ Mean = 50 (455)	51 (5–12, 178) ^15^ 56 (5–12, 142) ^72^ 34 (5–12, 117) ^19^ Mean = 48 (437)
13 to 17 years old Adolescent	14 (13–21, 590) ^15^ 20 (13–17, 148) ^72^ 33 (12–20, 22) ^14^ 25 (13–17, 190) ^19^ Mean = 18 (950)	26 (13–21, 590) ^15^ 31 (13–17, 148) ^72^ 24 (13–17, 190) ^19^ Mean = 26 (928)	60 (13–21, 590) ^15^ 49 (13–17, 148) ^72^ 57 (13–17, 190) ^19^ Mean = 58 (928)	42 (13–21, 590) ^15^ 51 (13–17, 148) ^72^ 25 (12–19, 193) ^13^ 49 (9–17, 88) ^64^ 46 (13–17, 108) ^19^ Mean = 41 (1,127)
18 to 39 years old Young adult	15 (22–35, 1,543) ^15^ 15 (18–35, 1,235) ^72^ 20 (16–39, 3,912) ^17^ 21 (18–35, 197) ^68^ 57 (18–39, 106) ^54^ 60 (18–64, 7,810) ^75^ Mean = 40 (14,803)	35 (22–35, 1,543) ^15^ 33 (18–35, 1,235) ^72^ 33 (18–39, 106) ^54^ 12 (18–64, 7,810) ^75^ Mean = 18 (10,694)	50 (22–35, 1,543) ^15^ 52 (18–35, 1,235) ^72^ 10 (18–39, 106) ^54^ 28 (18–64, 7,810) ^75^ Mean = 34 (10,694)	43 (22–35, 1,543) ^15^ 50 (18–35, 1,235) ^72^ 24 (16–39, 3,912) ^17^ 46 (18–35, 197) ^68^ 70 (18–39, 106) ^54^ Mean = 34 (6,993)
40 to 64 years old Adult	61 (36–64, 10,742) ^72^ 83 (≥40, 26,094) ^17^ Mean = 77 (36,836)	13 (36–64, 10,742) ^72^	26 (36–64, 10,742) ^72^	67 (36–64, 10,742) ^72^ 33 (≥40, 26,094) ^17^ Mean = 43 (36,836)
65 to 80 years old Older adult	79 (65–79, 17,071) ^72^ 81 (65–75, 7,261) ^75^ 86 (65–79, 9,609) ^75^ Mean = 81 (33,941)	9 (65–79, 17,071) ^73^ 7 (65–75, 7,261) ^75^ Mean = 8 (24,332)	12 (65–79, 17,071) ^72^ 12 (65–75, 7,261) ^75^ Mean = 12 (24,332)	71 (65–79, 17,071) ^72^ 67 (65–75, 7,261) ^75^ 35 (65–79, 9,609) ^77^ 51 (66–80, 8,919) ^74^ Mean = 58 (42,860)
>80 years old Elderly	79 (≥80, 9,095) ^72^ 83 (>75, 8,390) ^75^ 90 (80–89, 6,430) ^77^ Mean = 83 (23,915)	7 (≥80, 9,095) ^72^ 6 (>75, 8,390) ^75^ Mean = 7 (17,485)	14 (≥80, 9,095) ^72^ 11 (>75, 8,390) ^75^ Mean = 13 (17,485)	73 (≥80, 9,095) ^72^ 65 (>75, 8,390) ^75^ 31 (80–89, 6,430) ^77^ 52 (>80, 5,334) ^74^ Mean = 58 (29,249)

Data were extracted from multiple epidemiologic reports as cited. In many cases, our age bands do not strictly conform to other studies; therefore, data may be repeated and actual age ranges are provided for each data element. The dotted line represents the separation of children and young adults (0–39 years old) from older adults (>40 years old).

Since it is uncertain when the precise time of CA occurs, the no-flow times can be substantial. In our personal experience (since this is rarely reported), many of these children are noted to be cyanotic and lifeless by their parents when CPR is first begun, and epidemiologic reports confirm asystole as the most frequent presenting rhythm ^12, 13^. Young hearts fare much better than young brains as evidenced by return of spontaneous circulation (ROSC) rates of 30% or better ^12, 13^ (despite the lack of a witness in most cases), which are comparable to that of *witnessed* adult non-shockable OHCA ^18^. Infants were the only group in the large Japanese data set who had a lower incidence of ROSC ^19^. This may reflect a somewhat enhanced capacity of the heart to tolerate ischemia ^20^ compared with the brain. The fact that many arrests are indeed unwitnessed resulted in the observation that bystander CPR rates, though not dissimilar to those of other age groups, did not improve outcomes ^12, 13^. Presumably, CPR started by bystanders on younger patients is occurring later after the onset of no flow since a larger proportion of pediatric OHCA is unwitnessed (that is, the patient is found in no flow of uncertain duration). Bystander CPR initiated later in no flow likely lacks the benefits imparted by prompt bystander CPR in a randomized study of older adults ^21^.

The second important factor explaining why pediatric and young adult OHCA patients do not fare better is the extremely high incidence of asphyxial or respiratory etiology arrests as opposed to cardiac etiology. This is particularly true after infancy (when many congenital defects account for cardiac death) through young adulthood ( Table 3). Outcome also appears to vary on the basis of etiology, although this association is controversial. We previously found worsened neurologic outcomes and survival associated with non-cardiac etiology OHCA ^22^, which was likewise noted in the Korean Hypothermia Network (KORHN) registry ^23^, whereas the opposite association was noted by the ROC ^24^. Cardiac etiology OHCA tends to present with ventricular fibrillation and ventricular tachycardia, whereas non-cardiac etiology CA, such as asphyxia, often presents with pulseless electrical activity or asystole as a first rhythm ^25, 26^. Shockable rhythms are well known to yield the best outcomes ^23, 24, 27, 28^, even in children ^29^. In the recently reported pediatric hypothermia study (THAPCA-OHCA) ^30^, asphyxial OHCA accounted for more than 70% of all OHCA, and shockable rhythms were present in fewer than 10% of cases. This is compared with incidences of approximately 24% shockable rhythm in adult OHCA ^27, 28^. Young adults, who tend to make up the majority of overdose-related OHCA, generally present with non-shockable rhythms ^16^. Although it remains uncertain why asphyxial CA confers more severe outcomes, this observation has been consistently made in experimental comparisons of different forms of CA where insult time is matched ^31, 32^. Yet treatment of OHCA patients after ROSC is the same regardless of rhythm or etiology ^33^; the only difference is that coronary angiography is recommended in suspected cardiac etiology CA due to acute coronary occlusion ^16^.

The low incidence of pediatric OHCA relative to adult OHCA also means that paramedics are unlikely to have much repetition with resuscitation annually. With most things in medicine, “practice makes perfect”; therefore, low incidence means little practice. This is confounded by the added complexity resulting from pediatric life support ^34, 35^, which demands variable dosing and equipment sizing based on age and uses different algorithms than adult basic and advanced life support ^36, 37^. It is no surprise that in anonymous surveys paramedics report far less comfort with their technical skills in pediatric resuscitation and far less experience ^38^. Compliance with American Heart Association (AHA) guidelines for rate and depth is significantly improved in “adult size” adolescents compared with children who are 1 to 11 years old ^39^ achieving compliance rates comparable to that seen in adults within the same registry ^40, 41^.

In the past decade, the concept of developmental apoptosis has been identified and characterized ^42^. It appears that the cellular and molecular machinery used for normal pruning of neurons prior to birth and in infancy can be activated by sustained exposure to some sedatives or anesthetics (or both) with use consistent with what might be encountered with operating room or pediatric intensive care unit management or both ^43^. Impairment of neurogenesis may also be operating ^44^. The result of these developmental phenomena is that in children apoptosis may be accelerated, exceeding what is normally seen as a result of global ischemia-reperfusion injury and thus precipitating a more severe injury. This mechanism could contribute to poorer-than-anticipated outcomes after cardiac arrest in infants, although the upper age limit for potential contribution of this mechanism remains to be determined ^45, 46^. However, the quantitative contribution of this molecular disadvantage in infants to outcome after ischemic insults to the brain remains to be defined.

## Implications for management

The significant differences between the older adult and pediatric/young adult populations are important issues to consider for management. Since 2008, the AHA has recommended chest compression-only CPR (COCPR) for adults who have an OHCA that is witnessed by a bystander ^47^. This was based on pre-clinical and human observational data in adults that showed equivalent or improved outcomes when COCPR was used ^48– 53^. Pragmatically, COCPR is easier to teach and instruct (for 911 dispatchers) and results in fewer interruptions of CPR for rescue breaths as well as perhaps lower intrathoracic pressure (increasing coronary perfusion pressure). However, it must be noted that COCPR is ineffective in the pediatric population ^29^. In this study, COCPR had similar outcomes to standard CPR only among pediatric patients with arrests of cardiac origin, a distinct minority ( Table 3).

The AHA’s call to action is intended “for bystanders [and] does not apply to unwitnessed cardiac arrest, cardiac arrest in children, or cardiac arrest presumed to be of non-cardiac origin”, nor does it apply to paramedics who can provide full CPR ^47^. In practice, however, this distinction is not conveyed to the public or appreciated by 911 operators; we routinely care for asphyxial OHCA patients who have received COCPR. For children who had OHCA from non-cardiac causes, full CPR with rescue breathing yielded higher odds of ROSC, 1-month survival, and neurologically intact survival ^29^. When Ogawa and colleagues examined this issue in the same national database (Japan), they found a similar trend to better outcomes with full CPR (versus COCPR) in the 20- to 39-year-old cohort, which was fully explained by better outcomes in the non-cardiac etiology subset ^51^. Given the high prevalence of drug overdoses for arrest etiology in adolescents and young adults ^15, 54^, the now-dominant practice of instructing COCPR needs to be questioned if not modified to teach standard CPR in the younger population. Given that it is often easiest to teach a single approach and that outcomes from arrests of cardiac origin were not different in standard CPR versus COCPR, it would seem preferable to teach standard CPR for use across the full pediatric/young adult setting.

Differences in the characteristics of OHCA among patient populations are important when considering therapeutic hypothermia following OHCA. Two trials involving adult patients showed that therapeutic hypothermia improved neurologic outcomes in comatose survivors of OHCA with shockable rhythms compared with no temperature control ^55^ or relaxed normothermia ^56^. Interestingly, when comparing temperature management targeting 33°C versus 36°C after OHCA, the Targeted Temperature Management (TTM) trial found no significant difference in outcomes between groups ^57^. It should be noted that all of these studies recruited only patients with OHCA of presumed cardiac etiology ^55– 57^. In the underpowered (n = 260 compared with n = 939 in TTM) pediatric THAPCA-OHCA study, Moler and colleagues found a trend ( *P* = 0.14) toward benefit for hypothermia (33°C) compared with the normothermia arm (36.8°C) ^30^ with a number needed to treat of 12 and no increase in adverse outcomes. Compared with the adult studies, the pediatric THAPCA-OHCA study recruited primarily respiratory etiology (72%) non-shockable (>90%) OHCA. Present AHA guidelines ^33^ do not distinguish the temperature target for cardiac versus respiratory etiology OHCA ^33^, leaving open the option to select any value from 32°C to 36°C in adults. In pediatrics, the present guidelines ^34^ leave open the option to cool (32–34°C) or stay warm (36–37.5°C). Substantial pre-clinical evidence supports the benefits of deeper hypothermia (33°C) even when delayed for several hours in asphyxial CA ^58^. Given the existing data, one must question whether pediatric and young adult OHCA survivors, particularly those known to have had non-cardiac etiology OHCA, would not benefit from a lower temperature goal. When temperature strategies are compared ^30, 57^, it would appear that the potential benefits of cooling asphyxial arrest survivors outweigh the risks. Hypothermia has been suggested in pre-clinical studies to have specific protective effects against developmental apoptosis and this may also contribute to its efficacy in newborn asphyxia ^60^.

With regard to paramedic performance in the setting of pediatric arrests, recent studies have demonstrated the challenge for paramedic providers in the setting of OHCA in children given how rarely they are encountered in routine paramedic practice ^60^. However, simulation approaches applied systematically across multiple paramedic systems are being evaluated, as is the analysis of the specific errors that are most frequently observed in this setting ^61^.

## Conclusions

Given the unique facets of CA in children and young adults and the emerging importance of the opioid epidemic, we believe that a careful re-examination of our current care in regard to resuscitation training and temperature management is vital and that new investigation is crucial to optimizing outcomes in what should be a resilient population.
